# Altered Dynamics in the Circadian Oscillation of Clock Genes in Dermal Fibroblasts of Patients Suffering from Idiopathic Hypersomnia

**DOI:** 10.1371/journal.pone.0085255

**Published:** 2014-01-14

**Authors:** Julian Lippert, Hartmut Halfter, Anna Heidbreder, Dominik Röhr, Burkhard Gess, Mathias Boentert, Nani Osada, Peter Young

**Affiliations:** 1 University Hospital Muenster, Department of Neurology – Sleepmedicine and Neuromuscular Diseases, Albert-Schweitzer-Campus 1, Münster, Germany; 2 Institute of Medical Informatics, Albert-Schweitzer-Campus 1, Münster, Germany; University of Texas Southwestern Medical Center, United States of America

## Abstract

From single cell organisms to the most complex life forms, the 24-hour circadian rhythm is important for numerous aspects of physiology and behavior such as daily periodic fluctuations in body temperature and sleep-wake cycles. Influenced by environmental cues – mainly by light input -, the central pacemaker in the thalamic suprachiasmatic nuclei (SCN) controls and regulates the internal clock mechanisms which are present in peripheral tissues. In order to correlate modifications in the molecular mechanisms of circadian rhythm with the pathophysiology of idiopathic hypersomnia, this study aimed to investigate the dynamics of the expression of circadian clock genes in dermal fibroblasts of idiopathic hypersomniacs (IH) in comparison to those of healthy controls (HC). Ten clinically and polysomnographically proven IH patients were recruited from the department of sleep medicine of the University Hospital of Muenster. Clinical diagnosis was done by two consecutive polysomnographies (PSG) and Multiple Sleep Latency Test (MSLT). Fourteen clinical healthy volunteers served as control group. Dermal fibroblasts were obtained via punch biopsy and grown in cell culture. The expression of circadian clock genes was investigated by semiquantitative Reverse Transcriptase-PCR qRT-PCR analysis, confirming periodical oscillation of expression of the core circadian clock genes *BMAL1, PER1/2* and *CRY1/2.* The amplitude of the rhythmically expressed *BMAL1, PER1* and *PER2* was significantly dampened in dermal fibroblasts of IH compared to HC over two circadian periods whereas the overall expression of only the key transcriptional factor *BMAL1* was significantly reduced in IH. Our study suggests for the first time an aberrant dynamics in the circadian clock in IH. These findings may serve to better understand some clinical features of the pathophysiology in sleep – wake rhythms in IH.

## Introduction

Idiopathic hypersomnia comprises a combination of symptoms characterized by excessive daytime sleepiness and a constant decreased amount of alertness during daytime despite uninterrupted sleep pattern during the night. According to the diagnostic guidelines [1], a distinction is drawn between idiopathic hypersomnia with (>10 h) and without (<10 h) long sleep time.

Clinical diagnosis criteria are in concordance to narcolepsy. The diagnosis of idiopathic hypersomnia is made by excluding narcolepsy without cataplexy due to the lack of rapid eye movement (REM) sleep associated symptoms as early sleep onset REM phases in Multiple Sleep Latency Test (MSLT), sleep paralysis, hypnagogic hallucinations and fragmented sleep during the night. Further primary organic disorders have to be excluded such as psychiatric and neurological disorders, hormone dysregulation, use of drugs or substances, different genuine sleep disorders such as sleep-disordered breathing (e.g. obstructive sleep apnea syndrome (OSAS)), sleep-related movement disorders (periodic limb movement disorder, parasomnia), or sleep deprivation. Methodological diagnosis requires tools such as cardiorespiratory polysomnography (PSG), Multiple Sleep Latency Test (MSLT) and validated questionnaires as the Epworth Sleepiness Scale (ESS) [2]. Whereas excessive daytime sleepiness, called secondary hypersomnia, has a rather high prevalence of 4% to 6% of the general population [3] with a higher percentage of male because of a higher prevalence of sleep-disordered breathing, IH is still a rare disease of unknown prevalence and frequently overdiagnosed due to nosological uncertainties and the lack of epidemiological surveys [4].

Since IH can be clinically distinguished from narcolepsy without cataplexy [5]–[7] there is still a lack of normative data and a standardized procedure to classify the complex symptoms of IH concerning fatigue, decreased alertness, sleep pressure, depressive and mental co-morbidities and variations of circadian chronotypes. Up to now little is known to what extend the disturbance of the sleep-wake rhythm in IH is based on an altered interaction of environmental factors and possible genetic factors, namely the CLOCK genes. Recently, evidence for an impact of the *CRY2* gene on the phenotype of excessive daytime sleepiness in IH was presented by the analysis of segregation of a single nucleotide polymorphism (SNP) [8]. Over the last decade gene association studies extended the understanding of disorders affecting sleep and led to new diagnostic and therapeutic options. Associations between the HLA-system and circadian rhythm disorder, narcolepsy and REM-behavioural disorder as well as an association of restless legs syndrome with different genes had been identified using a genome wide association approach [9]. Conclusive genetic findings in idiopathic hypersomnia are still missing and are limited to rare epidemiological data, and unlike narcolepsy, a conclusive association between HLA-genotypes and IH could not be found [10].

It was postulated that a mismatch of the external environment to the internal master clock (e.g. jet lag, shift work etc.) or an alteration in the genetically based circadian clock as shown for the advanced or delayed sleep phase disorders are the two main reasons for circadian based sleep disorders [11].

Our individual daily behaviour and physiology is intrinsically regulated by a genetic clockwork controlling our daytime preference or chronotype [12], [13]. The identification of mutations in circadian clock associated genes in Delayed Sleep Phase Syndrome (DSPS) and Family Advanced Sleep Phase Syndroms (FASPS) has established the pivotal role of circadian rhythm in the pathogenesis of sleep disorders [14].

With respect to the symptoms of hypersomnia, we postulate a genetically determined circadian background of this sleep disorder as a major factor of the pathogenesis.

In mammals, the circadian master clock is located in the suprachiasmatic nucleus (SCN) in the brain hypothalamus. It is driven by a network of transcriptional feedback loops of circadian clock genes. Light input via the retinohypothalamic tract entrains the molecular circadian rhythm in the SCN and resets the circadian rhythm of peripheral clocks to daily light-dark cycle. The molecular mechanism of the circadian clock mechanism is composed of positive (*BMAL1,CLOCK*) and negative (*CRY1-2, PER1-3,REV-ERBα*) transcriptional feedback loops [15]. This mechanism is also active in peripheral tissues where it can be cell-autonomous and persists even in isolated cells [16], [17]. In peripheral tissues such as heart muscle cells, lymphocytes, keratinocytes or dermal fibroblasts the molecular-genetic network is under the control of the master clock in the SCN emanating signals such as hormone secretion to keep the peripheral oscillators synchronized [18]. The peripheral clocks can exist in cell-autonomous manner.

Previous studies suggests that these peripheral circadian oscillators mirror the individual chronotype i.e. the circadian period length and phase [19], [20].

In the present study we focused on a dermal fibroblast based assay to approach a possible aberrant circadian gene regulation in patients suffering from idiopathic hypersomnia.

## Methods

### Patients and controls

The study protocol, screening questionnaires and consent forms were approved by the Medical Ethical Committee of the University Muenster (ref.: 2009–361-f-S) and a signed written informed consent was obtained from each voluntary subject. Ten patients suffering from idiopathic hypersomnia (IH) were recruited from the department of sleep medicine University Hospital Muenster. They were diagnosed according to the ICDS2 criteria (Table 1). Average sleep latency in four consecutive MSLT had to be below eight minutes with no signs of sleep onset REM-phase. The Epworth Sleepiness Scale (ESS) as a useful instrument to measure the subjective daytime sleepiness had to be above >10 [21]. Patients with pre-existing illnesses such as restless legs syndrome, sleep disordered breathing, adiposity (BMI>35), heart- and neuromuscular diseases as well as anaemia and hypothyroidism (Table 1) as a cause of excessive daytime sleepiness were excluded. All patients underwent lumbar puncture for diagnostic purpose to exclude hypocretin deficiency or any signs of chronic inflammation in the central nervous system.

**Table 1:** Clinical characteristics of the study patients and age- gender matched controls with polysomnography findings and laboratory parameters.

sleep measures	idiopathic hypersomniacs	controls
Age	37,6±12,4	42,1±14,9
woman %	60,0	57,1
Epworth Sleepiness Score	14,5±3,2	8,2±1,8
MSLT min	4,6±1,6	
latency to sleep, min	12,2±10,2	
REM sleep %	16,6± 3,9	
N2 %	52,4±7,8	
N3%	19,8±8,5	
periodic legs movements, n/h	2,4±2,6	
apnea/hypopnea, n/h	1,0±1,1	
oxygen desaturation, n/h	0,6±0,6	
haemoglobin	13,9±1,4	
TSH mU/l	1,2±0,4	

Fourteen healthy unrelated and volunteer controls were included into the study. A modified version of the Horne-Ostberg Chronotype Questionnaire (AutoHCQ) was applied to exclude extreme chronotypes among the control population with an average HOQ score of 46±8 [22], [23]. Shift or night work, severe and chronic illnesses and intake of any medication and cognitive modifying drugs were exclusion criteria.

Dermal fibroblasts were obtained from a 2mm punch biopsy of the anterior pelvic region and grown in cell culture dishes containing Dulbecco's modified Eagle medium, supplemented with 20% fetal bovine serum (FBS), 1% Penicillin-Streptomycin-Neomycin antibiotic mixture and 1% glutamate.

Each cell line was seeded on fourteen 2cm dishes to extract the total RNA at various time points (0,3,6,12,24,27,30,36,48,51,54,60,66,72 hours) after addition of dexamethasone. As cell passage affects significantly the circadian clock gene expression, all cell lines were in the 6^th^^+^/−1 passage [24]. Before initiating the circadian rhythm of the fibroblasts with dexamethasone (100nm), the cells were starved for 48h in a serum free ‘Defined fibroblast maintenance medium’ (provitro) to reduce external cues e.g. serum factors and hormones, which crucially affect the molecular properties of the circadian clock such as period length, amplitude and phase [25]. After dexamethasone (100nm) treatment for 30min – defined as time 0– the cells were returned to serum free medium. The isolation of the total RNA at the indicated times was performed using TRIzol® Reagent (invitrogen) followed by the QIAGEN RNeasy Mini Kit (Qiagen, Hilden, Germany). Applying the QuantiTect® Reverse Transcription Kit (Qiagen, Hilden, Germany) the total RNA was reverse transcribed into cDNA.

Quantitative real-time polymerase chain reaction (qRT-PCR) was performed with SYBR Green PCR technology determining the gene expression profile of the circadian rhythm genes *BMAL1 (ARNTL1), CRY1, CRY2, PER1, PER2*. The ribosomal RNA *18S* served as a house keeping gene (h18S) [26]. When analyzing the circadian gene expression profile we focused mainly on two aspects:

(I) we examined the dynamics in the gene expression profile namely the up and down regulation on the transcriptional level over two periods of 24h and
(II) the overall gene expression rate at the indicated time points in both groups.

Due to a gradual decrease of circadian clock gene expression over a period of three days after glucocorticoid shock, we examined the course of the gene expression separately in two consecutive 24h periods – 1^st^ period starting 6h after dexamethasone shock to 30h, 2^nd^ period starting at 30h to 54h.

### Examination of gene expression profile and mathematical model

For calculating the relative expression of the genes of interest we employed the software tool (REST©) [27]. We determined the individual circadian gene expression profile for each subject defining time point ‘0’ as the reference condition.

To demonstrate the periodic circadian oscillation of the clock genes over two consecutive periods with 24 hours (1^st^ 6h-30h, 2^nd^ 30h–54h), sine curves were fitted to the actual measurements at the different time points by the nonlinear least-squares method. The mathematic analysis was performed by Texas Instruments® software.

As the data of the experimental measurements are underlying inherently large fluctuations and errors we chose a method which is proven in a clinical setting to predict rhythmic variations of gene expression despite a small number of samplings.

This method is based on regression techniques and it is suitable to analyze the periodicities of unequidistant measurements [28]. To achieve a high accuracy in predicting the rhythmic gene oscillation, we applied the procedure of multiple component analysis [29].

Three sine curves (C = 3) were fitted to six experimental measurements in each case by least squares method. The first sine curve is fitted to the actual measurements at time points 3h, 6h, 12h, 24h, 27h and 30h, the second 6h, 12h, 24h, 27h, 30h, 36h and the third one 12h, 24h, 27h, 30h, 36h, 48h in the 1^st^ period – (2^nd^ period see SI).

The multiple component procedure is based on the following function with three (C = 3) fixed anticipated periods,
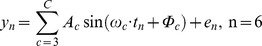
where y_n_ indicates gene expression level at time t_n_, C is the number of sinusoidal components, A_c_ indicating the amplitude of sine curve, ω_c_ is the angular frequency, i.e. ω_c_  = 2π/τ_c_, Φ_c_ is the acrophase and e_n_ indicating the offset value.

The accuracy of fit is indicated by root mean square errors, i.e. the squares of differences between the actual measurements and the estimated best-fitting sinus curve.

For the considered period we obtained a best fit sine curve which is defined as the average value of the three rhythmic sine functions fitted to the experimental data.

Focusing on the overall amplitude defined as half the difference between the maximum and minimum of the average of multiple sine curves, we applied the non-parametric pairwise comparison of Friedman's analysis to compare the up and down regulation of circadian clock genes on a logarithmic scale between the two study groups.

### Direct comparison of the overall gene expression rate

As the above mentioned analysis does not provide evidence about the overall transcriptional levels of circadian clock genes in each study group, we examined separately the relative mRNA quantity at each time point of measurement applying the ΔΔC_t_-method. Comparison of the expression rate of the circadian clock genes *BMAL1, CRY1/2, PER1/2* between the two groups was performed by applying the non-parametric Mann–Whitney–Wilcoxon test.

Asymptotic significances (2-sided tests) are displayed. The significance level is P<0.05. At each time point data are presented as the mean ± standard error of the mean (SEM).

## Results

In order to determine the circadian rhythm of fibroblasts from hypersomniac patients, we performed mRNA expression analysis of several known circadian clock genes. RNA was analyzed from fibroblast cells after treatment of the cells with dexamethasone (time point ‘0’) for initiation of the circadian cycle. The mRNA amounts of each gene of interest were determined by qRT-PCR relative to *18S* mRNA for normalization.

**Figure 1:**
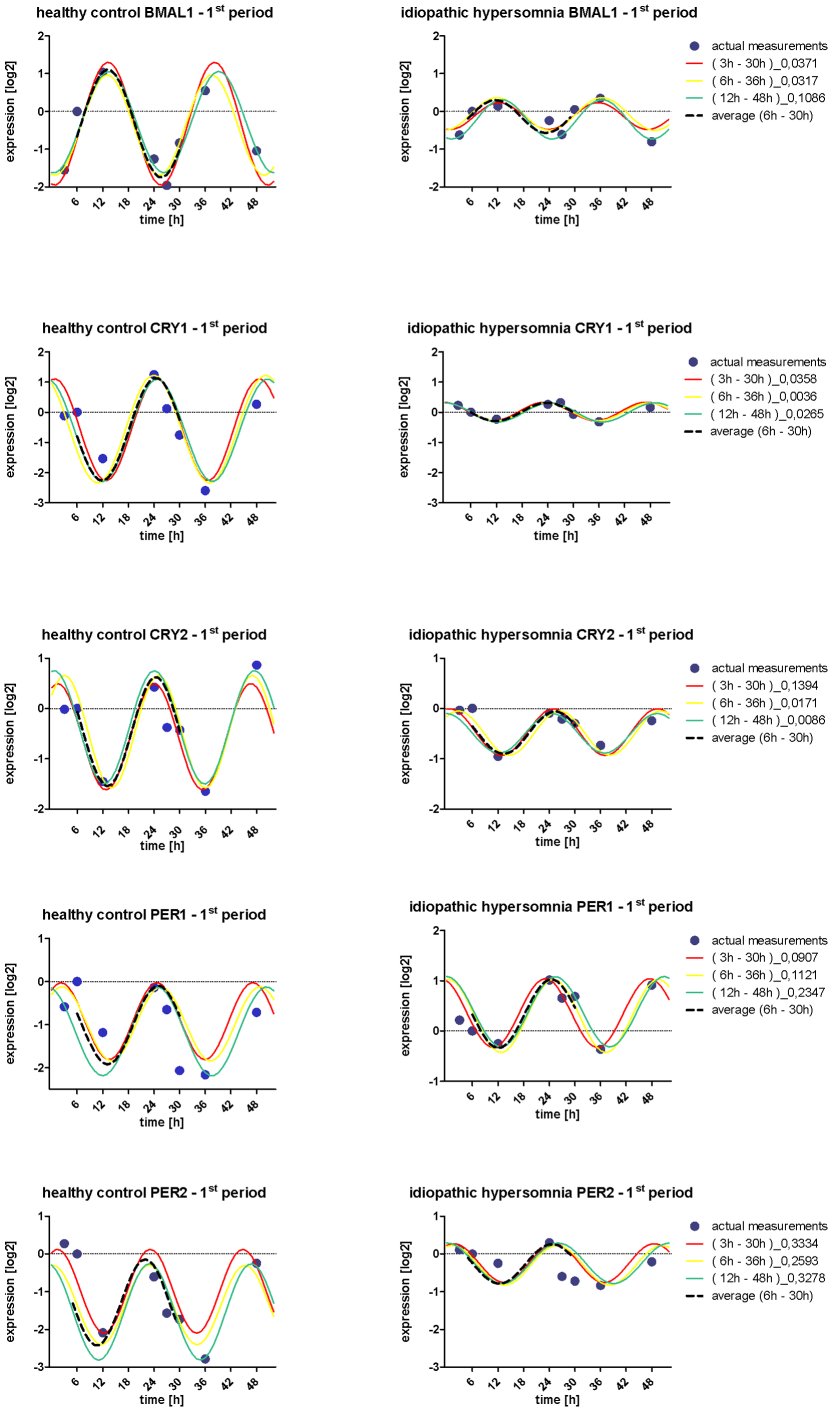
Using total RNA from primary dermal fibroblasts collected at the indicated time points were used to perform real-time PCR to measure the expression of circadian clock genes. Blue dots indicate actual measurements, the colored lines represent the gene expression profile relative to *18S* rRNA in the shape of a sine curve to prove circadian gene expression in all examined cell lines. Each colored line indicates an approximated sine wave by least squares method to 6 actual measurements (I) (3h, 6h, 12h, 24h, 27h, 30h), (II) (6h, 12h, 24h, 27h, 30h, 36h), (III) (12h, 24h, 27h, 30h, 36h, 48h). The applied method is based on the multiple components analysis which allows fitting several significant functions to the experimental data. The broken black line indicates the best fit sine curve defined as the average value of the three rhythmic functions fitted to the data. Numbers in parentheses beside the figures indicate the time points used for prediction and numbers at the right indicate root-mean-square errors (MSE). The smaller the MSE, the more accurate is the prediction of rhythmic circulation. Given examples show exemplarily the harmonic expression in two fibroblast cell lines from one healthy control (left) and one idiopathic hypersomniac (right) depicting the flattened circadian amplitude of gene expression profile in the IH group versus healthy control.

The predicted sine curves fitted to the actual measurements by the nonlinear least-squares method (Figure 1) as described above represent the individual gene expression profile in dermal fibroblasts in each individual in both groups.

As the oscillation of the circadian gene expression dampens constantly over the course of 72h, the two consecutive periods -1^st^ period starting 6h–30h (Figure 1), 2^nd^ period starting 30h–54h (Figure S1)- were statistically analyzed separately.

Though the number of the actual measurement over the period of 72h is too small to identify significant differences in phase and period length between the two study groups, the sine curves display the inter-individual dynamics in the gene expression as differences in amplitudes.

We calculated the dynamics of the transcriptional up- and down regulation of the circadian rhythm genes on a logarithmic scale independent of the basal transcriptional level. Statistical analysis was performed using the SPSS Software, version 18.0.0. (07/30/2009; SPSS, Inc., Chicago, USA). Categorical variables are expressed as frequency and percentage, whereas continuous variables are presented as mean ± SD. Before statistical testing, each continuous variable was analyzed for its normal distribution using the Kolmogorov-Smirnov test. The actual measurements of the first– and second period as the nonparametric time-course dependent variables were assessed using Friedman test. Pairwise multiple comparisons following the Friedman Test were performed using the procedure proposed by Dunn.

The Mann-Whitney-U Test was used to compare the nonparametric variable between two independent study groups - healthy controls and idiopathic hypersomniacs. The Wilcoxon Test was used to compare the nonparametric variable between two dependent study groups. Differences were considered significant at P<0.05.

We analyzed the overall transcriptional rate of each single time point over 72h. In each examined individual of both study groups the differences of the gene expression rate of *BMAL1, CRY1, CRY2, PER1* and *PER2* on a logarithmic scale between each consecutive time point was significant applying the Friedman Test. This indicates a clear and repetitious cycling of peripheral circadian gene expression in all examined subjects.

**Figure 2:**
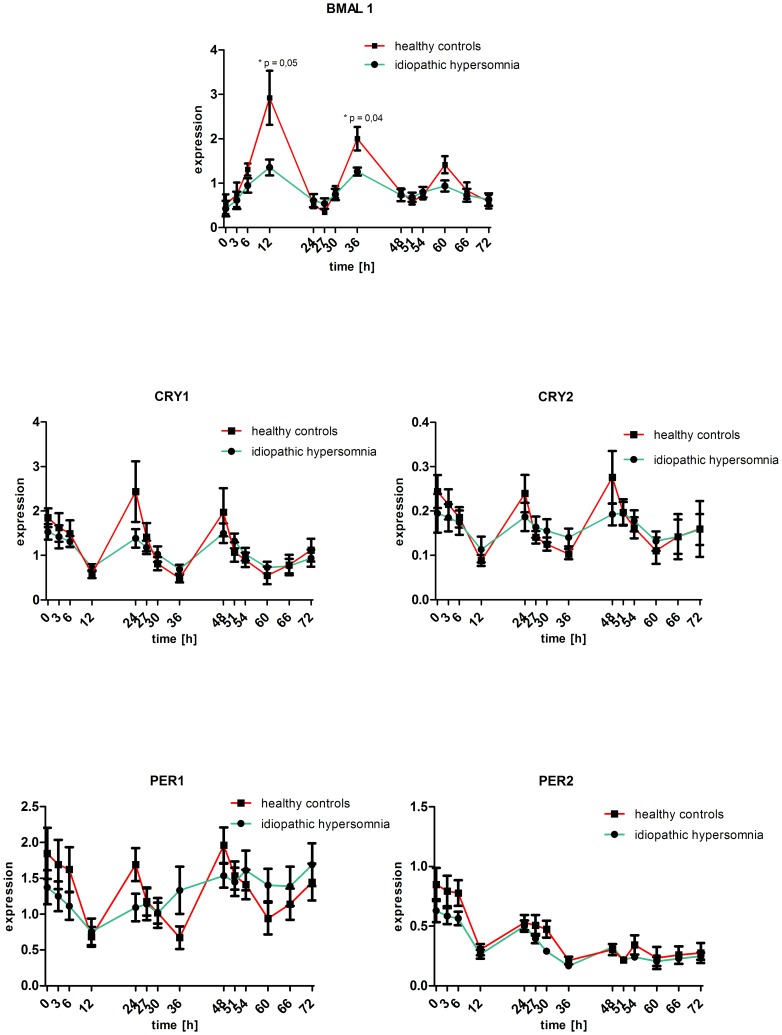
A direct comparison of the overall gene expression of the core circadian clock genes at the indicated time points reveal no significant difference in the absolute amount of gene expression with exception in case of *BMAL1* at time point 12h (P = 0.05) and 36h (P = 0.04). At these points the overall expressional rate in the group of healthy controls is significantly higher compared to the patient cohort. The x-axis reflects the actual interval of the sampling points. The black dots indicate the averaged overall gene expression values ± SEM of both study groups over the course of 72h.

Comparing the averaged overall transcriptional rate of all individuals in both study groups at each time point of measurement over two periods there is no significant difference between the two study groups except in case of *BMAL1* at time point 12h – reduced in the group of idiopathic hypersomniacs by 62% (P = 0.05) and 36h – reduced by 59% (P = 0.04) (Figure 2).

**Figure 3:**
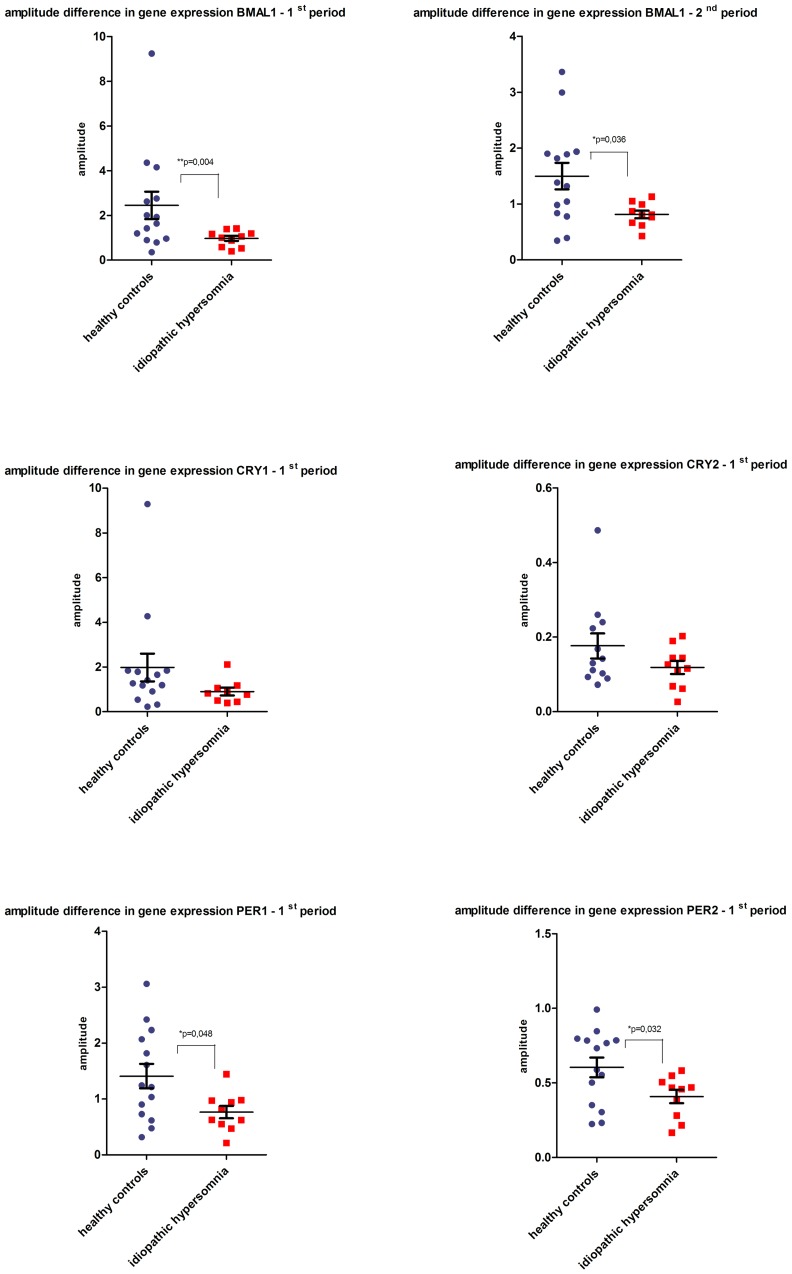
Comparison of the individual overall amplitude difference of circadian gene expression rate during the 1^st^ 24h-period starting 6h-30h and in case of *BMAL1* during the 2^nd^ 24h-period starting 30h-54h between healthy controls and idiopathic hypersomniacs. On the y-axis dots indicate for each examined individual the overall amplitude defined as the half difference between maximum and minimum of the average value of the multiple sine curves. Black lines indicate the averaged values ± SEM considering P<0.05 as significant. *BMAL1* reveals the strongest damping in circadian gene oscillation in the group of idiopathic hypersomniacs in comparison to healthy controls over two consecutive 24h-periods. In case of *PER2* and *PER1* the overall amplitude in the group of idiopathic hypersomniacs is significantly dampened in the 1^st^ 24h-period, in the 2^nd^ period there is no significant damping (data not shown). Though *CRY1* also shows a clear diminished oscillation in the patient group, the difference remains not significant due to a high SEM whereas *CRY2* shows no significant amplitude difference between the two study groups.

Concerning the circadian amplitude composed of the difference between peak and nadir of the circadian gene expression we detected a highly significant damping in the cycling expression of the three clock core components *BMAL1, PER1* and *PER2* between the two study groups in the first period and in case of BMAL1 also in the second period (Figure 3). The calculation of the differences in the amplitudes defined as the half differences between the maximum and the minimum is based on the average value of the multiple sine curves (Figure 1 and Suppl. Figure 1).

When analyzing this aspect we applied the Mann-Whitney-Test indicating an exact two sided significance. The amplitude of *BMAL1* expression in the group of idiopathic hypersomniacs is significantly reduced by 63% (P = 0.004, Figure 3) in the first period and reduced by 46% (P = 0.036, Figure 3) in the second period compared to healthy controls.

The amplitude of *PER1* is reduced by 45% (P = 0.048, Figure 3) in the 1^st^ period compared to the control group and so is the amplitude of *PER2* reduced by 32% (P = 0.032, Figure 3) in the 1^st^ period. The two further analyzed amplitudes of circadian rhythm genes *CRY1* is not significant (P = 0.064) due to a high SEM in the 1^st^ period and the amplitude difference of *CRY2* between the two study groups shows also no significant difference in both examined periods.

## Discussion

The complex of symptoms of idiopathic hypersomnia suggests a disturbed chronobiology as one of the heterogeneous aspects of this sleep disorder. In contrast to narcolepsy, the pathophysiology and pathogenesis of idiopathic hypersomnia remains largely unknown. As no animal models are available so far, the experimental approaches are limited.

In this study we chose an in vitro model which allowed us to measure individual patterns of clock gene expression in primary fibroblasts. Brown *et al. (2008)* [12] demonstrated that primary cells from skin biopsies are ideal for surrogate measurements for assessing circadian phenotypes as these cells demonstrate a cell-autonomous circadian rhythm in cell culture.

Starving primary cells in serum free fibroblasts maintenance medium under constant environmental conditions as described above and initiating circadian rhythms by dexamethasone enabled us to describe precisely the differences in the dynamics of circadian clock gene expression over two periods of 24h between the two study groups by RT-PCR. As numerous studies reveal the crucial effect of hormonal factors on interindividual differences of circadian clock properties such as phase, period length and circadian gene expression rate [25], [30], we minimized this influence by using a standardized medium depleted of serum factors.

All fibroblast cell lines from idiopathic hypersomniacs and healthy controls included in this study showed rhythmic expression patterns of circadian clock genes leading to a harmonic regression function. Due to a limited number of measurements of total mRNA expression over the two periods, an exact prediction and comparison of period length remained statistically imprecise. Nonetheless we have been able to display the reciprocal expression pattern of each examined circadian clock gene in all fibroblast cell lines in accordance to the model of positive and negative transcriptional feedback-loops.

We observed significantly dampened overall amplitude of the expression values of the circadian clock core-gene *BMAL1* as the difference between peak and nadir over two periods in fibroblasts obtained from idiopathic hypersomniacs in comparison to healthy controls. The overall amplitude of the clock genes *PER1* and *PER2* were also significantly reduced in the first period. In the second period the difference was not significant (P = 0.07) which could be rather explained by the constant dampening of the gene expression amplitude over several periods after glucocorticoid or serum shock. This effect is additionally increased in serum free conditions.

Though the amplitude in gene expression profile is significantly decreased, the overall expression level of the cycling gene at each time of measurement across the two periods is not. These findings suggest a diminished turnover and degradation of the circadian clock core components affecting the dynamics of the molecular circadian network. Despite a relatively small number of samples in which subtle differences of investigated circadian rhythm associated components may have gone unnoticed due to limited statistical power, our study provides evidence of a significant reduction in the amplitude of the key transcriptional factors *BMAL1, PER1* and *PER2* of the molecular clock system. The amplitude of expression of the *CRY1* gene was not significant though most IH patients exhibited a low *CRY1* amplitude. A larger cohort of patients might lead to more significant data in the future. Moreover, circadian expression of *CRY1* has been shown to be delayed in relation to the peak expression of other circadian genes [31] while in the present study the regulation of expression of the various circadian clock genes is only slightly different which indicate to a different mode of regulation in the human fibroblasts.

Over the last decades numerous studies have revealed the impact of the circadian deregulations in mood spectrum disorders raising the question of a differential diagnosis between selfsame and idiopathic hypersomnia as well as circadian rhythm sleep disorders particularly sleep phase delayed syndrome [32]–[41].

We propose that a dampened amplitude and an altered dynamics of circadian clock genes which influence transcriptional processes of further downstream clock-controlled genes [42], contribute to the pathogenesis of idiopathic excessive daytime sleepiness.

It has been well established that deletion or mutations of core clock genes leads to alterations in the sleep behavior, sleep architecture and sleep amount indicating that the expression of most of these genes is required for the maintenance of sleep-wake cycles. Deletion of *BMAL1* which showed the most obvious deregulation in the expression pattern in our study leads to arrhythmicity of locomotor activity [43]. A detailed analysis of the sleep-wake behavior of *BMAL1/Mop3^−/−^* mice showed significant more overall total sleep time, NREM and REM sleep in comparison to the wild type [44]. Interestingly, the sleep time is increased in *BMAL1/Mop3^−/−^* knockout mice in analogy to the clinical findings in patients with IH. Therefore deregulation of *BMAL1* gene as a common element participating in both circadian and sleep regulatory processes might be an indicator for idiopathic hypersomnia. On the other hand it had been shown recently that an unnatural sleep-wake cycle affects the expression of certain genes including core clock genes [45]. Currently we are at the beginning to understand the interaction of aberrant circadian clock genes with sleep architecture and –duration.

However, the expression of the circadian genes is regulated at various levels. Epigenetic, transcriptional as well as posttranscriptional mechanisms contribute to the complex regulation observed under various conditions [46]. Recent investigations on the regulation of the circadian rhythms have confirmed the point that not only transcriptional regulation is important for the circadian rhythm but also posttranscriptional mechanisms [47], [48]. The latter could show that a temperature shift of cycling cells to lower temperatures yielded in significant lower amplitudes of *BMAL1* promoter activity [49].

Whether the changes in the expression amplitude observed here were due to transcriptional or posttranscriptional mechanisms of the consequence of a different mode of regulation remains to be shown.

Consistent with our findings and hypothesis, we followed an approach of a previous study examining the circadian rhythm of melatonin in idiopathic hypersomniacs with long sleeping time and signs of sleep drunkenness upon awakening. This study reveals a phase delayed circadian rhythm of melatonin with a lower nighttime melatonin concentration compared to the control subjects [50]. As a small number of neurons of the SCN control the pineal melatonin secretion via a polysynaptic pathway [51], it is thought that a dampened oscillation of circadian clock genes as described above effects the decreased melatonin concentration in IH. This hypothesis is strengthened by the fact that lesions in the region of the central pacemaker reduce the amplitude of circadian rhythms of sleep-wakefulness, spontaneous locomotor activity and body temperature profoundly [52].

Despite the advantage of using primary fibroblasts as surrogate measurements for the central clock [53], we are aware of certain limitations of this fibroblast based model; the genetically based hypothalamic pacemaker is modified by various environmental cues such as temperature [54]–[56], metabolic status [57]–[59], behavioral activity rhythm [60] and projections from different brain regions like brain stem and cortex [61] which additionally influence diurnal pattern of behavior. Following this idea, future studies mainly based on the two-process model of sleep regulation are needed to evaluate individual circadian phenotypes in a clinical context leading to personalized therapy options [62].

In summary, the present study is the first one to correlate directly the heterogeneous clinical symptoms of the primary sleep disorder of idiopathic hypersomnia with the genetically determined circadian clock machinery apart from the melatonergic and hypocretinergic system. This offers both a new approach for a better understanding of the pathophysiology of this sleep disorder and possibly new options for pharmacological interventions [63].

## Supporting Information

Figure S1 **Clock gene expression in dermal fibroblasts reflects individual circadian rhythm of each individual.** Figures on the left show circadian clock gene expression of one healthy control and on the right of one idiopathic hypersomnia exemplarily during the second period starting 30h – 54h after dexamethasone shock. Applying the multiple components analysis the colored sine curves were fitted to the 6 actual measurements (I) (27h, 30h, 36h, 48h, 51h, 54h), (II) (30h, 36h, 48h, 51h, 54h, 60h), (III) (36h, 48h, 51h, 54h, 60h, 66h) by least squares method. The average value of the three rhythmic functions is indicated by the broken black line. Numbers in the parentheses indicate the time points for prediction and besides root mean square errors.
(TIF)
