# Supplementary figures and images for: Altered Dynamics in the Circadian Oscillation of Clock Genes in Dermal Fibroblasts of Patients Suffering from Idiopathic Hypersomnia
Source: PLoS One. 2014 Jan 14;9(1):e85255. doi: 10.1371/journal.pone.0085255 (PMC3891749; doi:10.1371/journal.pone.0085255)

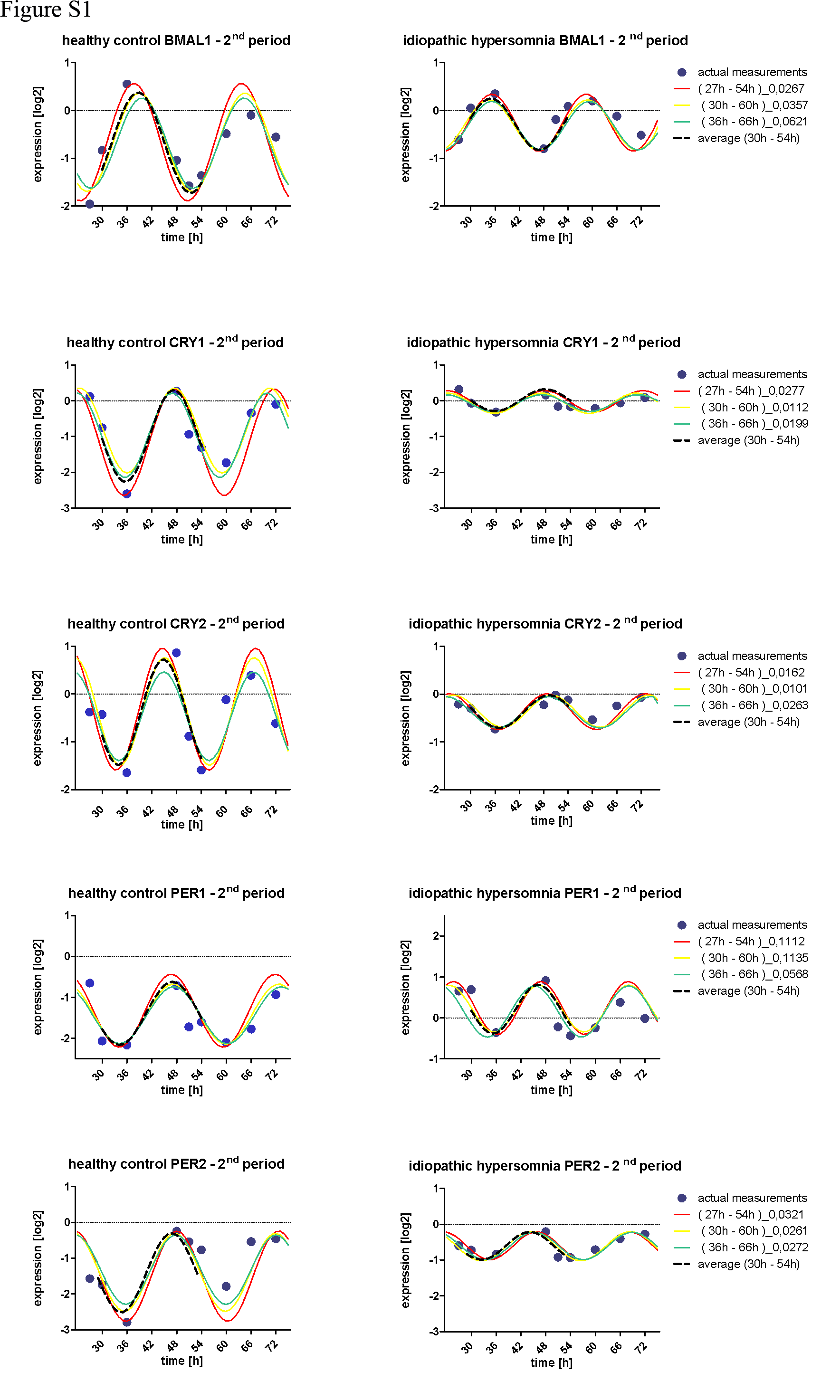

Supplement: Figure S1 — Clock gene expression in dermal fibroblasts reflects individual circadian rhythm of each individual. Figures on the left show circadian clock gene expression of one healthy control and on the right of one idiopathic hypersomnia exemplarily during the second period starting 30h – 54h after dexamethasone shock. Applying the multiple components analysis the colored sine curves were fitted to the 6 actual measurements (I) (27h, 30h, 36h, 48h, 51h, 54h), (II) (30h, 36h, 48h, 51h, 54h, 60h), (III) (36h, 48h, 51h, 54h, 60h, 66h) by least squares method. The average value of the three rhythmic functions is indicated by the broken black line. Numbers in the parentheses indicate the time points for prediction and besides root mean square errors. (TIF) [file pone.0085255.s001.tif]
